# Data on spatiotemporal land use land cover changes in peri-urban Addis Ababa, Ethiopia: Empirical evidences from Koye-Feche and Qilinto peri-urban areas

**DOI:** 10.1016/j.dib.2017.04.018

**Published:** 2017-04-20

**Authors:** Messay Mulugeta, Bechaye Tesfaye, Addis Ayano

**Affiliations:** a College of Development Studies, Addis Ababa University, Ethiopia; b World Vision-Ethiopia, Addis Ababa, Ethiopia

**Keywords:** Land use, Land cover, Peri-urban, Koye-Feche, Qilinto, Addis Ababa

## Abstract

Urban expansion is one of the key problems in Ethiopia resulting in displacement of the rural people inhabiting areas bordering the cities/towns. It is also resulting in land use land cover (LULC) changes affecting the livelihoods of the people and the ecosystems [1], [2]. The data presented in this article, therefore, shows the spatiotemporal LULC changes of peri-urban expansion areas known as Koye-Feche and Qilinto, around Addis Ababa City (the capital of Ethiopia). The data were generated from Landsat Thematic Mapper (TM) and Enhanced Thematic Mapper Plus (ETM+) images (with path/row numbers 168/054) by using ERDAS EMAGINE 2014 software. The precision of the images was verified by geolocation data collected from ground control points by using Geographic Positioning System (GPS) receiver. The data indicate that the built-up areas have increased by 1017.85 ha (10.178 km^2^) with 89.1%, 58.4%, 47% and 13% decline of plantation (mostly eucalyptus woodlots), grasslands, riverine vegetation (forestland) and cropland, respectively, between 1986 and 2016.

**Specifications Table**

**Table:** 

Subject area	Geography and Environmental Studies
More specific subject area	Land use land cover change, urban sprawl
Type of data	Table, figure and text file
How data was acquired	Data were extracted from Landsat TM and Landsat ETM^+^ images with path/row numbers 168/054 and firsthand data were acquired by using GPS-based ground survey technique.
Data format	Analyzed
Experimental factors	
Experimental features	The images were geo-referenced with World Geodetic System (WGS) 1984 datum and Universal Transverse Mercator (UTM) projection system zone 37 North. The images were classified based on visual interpretation and supervised classification using ERDAS EMAGINE 2014 software.
Data source location	Landsat and Koye-Feche and Qilinto areas ( 8°52′–8°56′N, 38°48′–38°52′E)
Data accessibility	The data is with this article

**Value of the data**

• The data is helpful to Addis Ababa City administrators to speculate the extent of the spatiotemporal expansion of Addis Ababa and its potential impacts on the surrounding areas.
• The data provides information on the status of urban expansion towards rural peri-urban areas around Addis Ababa.
• The data is vital to model urban expansion towards rural peri-urban areas surrounding Addis Ababa to mitigate its adverse impacts on the livelihoods of the people inhabiting the area and the ecosystem.
• The data is useful to researchers, urban planners and experts working in the field.

## 1 Data

**Fig. 1:**
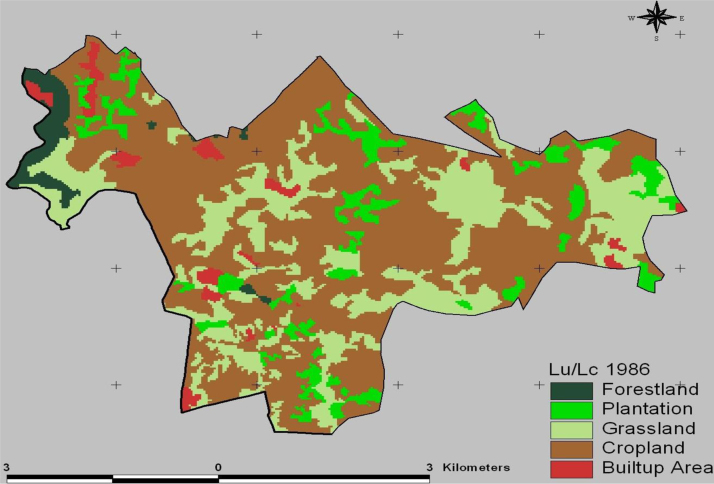
LULC classes of Koye-Feche and Qilinto areas in 1986.

**Fig. 2:**
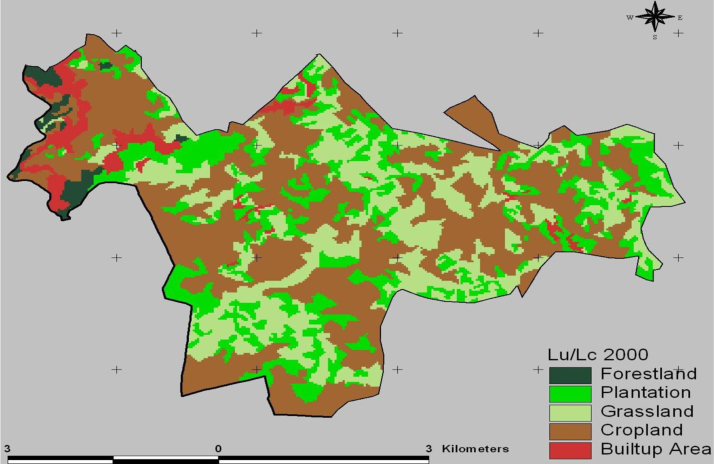
LULC classes of Koye-Feche and Qilinto areas in 2000.

**Fig. 3:**
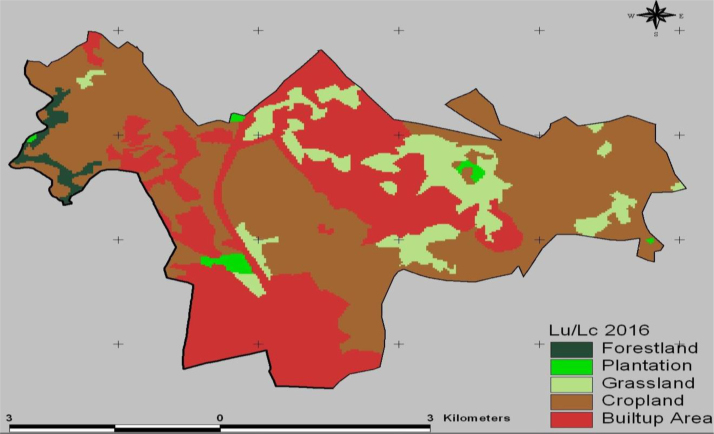
LULC classes of Koye-Feche and Qilinto areas in 2016.

The data in this article provides information on the spatiotemporal LULC changes in Koye-Feche and Qilinto urban expansion areas around Addis Ababa City between 1986 and 2016. Fig. 1, Fig. 2, Fig. 3 illustrate pictorially the spatiotemporal LULC classes of the area in 1986, 2000 and 2016. Cropland and grassland had dominated the land use in 1986 (Fig. 1) with very few built up areas, plantation and forestland. In 2000 (Fig. 2), plantation was tremendously expanded and cropland was considerably reduced. In 2016 (Fig. 3), built up area was extremely enlarged, cropland was almost disappeared and some part of the cropland was replaced by built up areas. Table 1 demonstrates LULC extent in hectare and percentage in 1986, 2000 and 2016 as well as rate of LULC changes in hectare (ha) and percentage (%). Table 2, Table 3, Table 4 demonstrate LULC change matrix between 1986 and 2000, 2000 and 2016, and 1986 and 2016. Table 5 illustrates rate of LULC change in hectare per year.

## 2 Experimental design, materials and methods

Landsat Thematic Mapper (TM) and Enhanced Thematic Mapper Plus (ETM+) images (with path/row numbers 168/054) as well as GPS-based ground survey records were vital data sources for this data article. The analyses, such as data extraction and LULC classification, were done by using ERDAS IMAGINE Version 2014 software. The images were geo-referenced with World Geodetic System (WGS) 1984 datum and Universal Transverse Mercator (UTM) projection system zone 37 North. Supervised and unsupervised image classifications techniques were employed to extract the data [3]. Supervised classification involved selecting pixels that represent land cover classes that are recognized by the analyst. Unsupervised image classification is more computer-automated. It enables the analyst to specify some parameters that the computer uses to reveal statistical patterns that are inherent in the data. These patterns are simply clusters of pixels with similar spectral characteristics. Due to similar spectral characteristics of grass, crop and bush lands, which were determined to be independent classes before classification, the application of unsupervised classification may not give good results. As a result, in the data extraction process, supervised image classification was used. After determining the land use features, the next step was LULC change detection. This was done through overlaying the classified satellite imageries and analyzing by image differencing algorithm. Finally, the outputs of image classifications were verified by conducting ground truth while recording *x* and *y* coordinates of the sample spatial features using GPS. Depending on the scope of the study and visual interpretation of the satellite imageries, five classes were identified in Koye-Feche and Qilinto peri-urban areas. These are forestland (including riverine bushes and shrubs), plantation (mostly eucalyptus woodlots), grassland, cropland and built-up area (including any sort of housing construction, road and other infrastructures) in the locality.

## Figures and Tables

**Table 1:** LULC extents and changes in Koye-Feche and Qilinto areas 1986–2016.

LULC classes	1986		2000		2016		Change 1986–2000		Change 2000–2016		Change 1986–2016	
	ha	%	ha	%	ha	%	ha	%	ha	%	ha	%
Forestland	89.6	2.9	58.1	1.8	47.5	1.5	−31.6	−35.2	−10.5	−18.1	−42.1	−47.0
Plantation	278.9	8.9	676.7	21.5	30.4	1.0	397.8	142.6	−646.3	−95.5	−248.3	−89.1
Grassland	820.8	26.1	812.8	25.9	341.6	10.9	−8.0	−1.0	−471.2	−58.0	−479.2	−58.4
Cropland	1860.7	59.2	1450.9	46.2	1612.6	51.3	−409.8	−22.0	161.73	11.1	−248.0	−13.3
Built up	90.5	2.9	142.1	4.5	1108.4	35.3	51.6	57.0	966.2	680	1017.8	1124.6
**Total**	**3140.6**	**100**	**3140.6**	**100**	**3140.6**	**100**	**0.0**	**0.0**	**0**	**0.0**	**0**	**0.0**

**Table 2:** LULC change matrix between 1986 and 2000.

LULC classes	Forestland		Plantation		Grassland		Cropland		Built-up area		Total	
	ha	%	ha	%	ha	%	ha	%	ha	%	ha	%
Forestland	35.6	39.8	0.0	0.0	17.6	2.1	4.2	0.2	0.5	0.6	58.1	1.8
Plantation	2.6	2.9	94.8	34.0	167.9	20.5	389.3	20.9	22.05	24.4	676.7	21.5
Grassland	5.4	6.0	87.8	31.5	265.5	32.3	446.6	24.0	7.47	8.3	812.8	25.9
Cropland	24.7	27.5	89.2	32.0	346.1	42.2	939.5	50.5	51.48	56.9	1450.9	46.2
Built-up	21.3	23.8	7.1	2.5	23.7	2.9	81.0	4.4	9	9.9	142.1	4.5
**Total**	**89.6**	**100**	**278.9**	**100**	**820.8**	**100**	**1860.7**	**100**	**90.5**	**100**	**3140.6**	**100**

**Table 3:** LULC change matrix between 2000 and 2016.

LULC classes	Forestland		Plantation		Grassland		Cropland		Built-up area		Total	
	ha	%	ha	%	ha	%	ha	%	ha	%	ha	%
Forestland	24.5	42.2	0.5	0.1	1.5	0.2	10.2	0.7	10.8	7.6	47.5	1.5
Plantation	0.6	1.1	1.7	0.3	7.8	1.0	20.3	1.4	0	0.0	30.42	1.0
Grassland	0.0	0.0	36.5	5.4	138.0	17.0	159.5	11.0	7.65	5.4	341.64	10.9
Cropland	31.4	54.1	351.5	51.9	335.4	41.3	799.7	55.1	94.59	66.6	1612.62	51.3
Built-up	1.5	2.6	286.5	42.3	330.0	40.6	461.3	31.8	29.07	20.5	1108.35	35.3
**Total**	**58.1**	**100**	**676.7**	**100**	**812.8**	**100**	**1450.9**	**100**	**142.11**	**100**	**3140.6**	**100.00**

**Table 4:** LULC change matrix between 1986 and 2016.

LULC classes	Forestland		Plantation		Grassland		Cropland		Built up area		Total	
	ha	%	ha	%	ha	%	ha	%	ha	%	ha	%
Forestland	36.0	40.2	0.0	0.0	11.5	1.4	0.0	0.0	0.0	0.0	47.5	1.5
Plantation	2.5	2.8	3.9	1.4	13.2	1.6	7.9	0.4	2.88	3.2	30.42	1.0
Grassland	1.4	1.5	12.9	4.6	146.5	17.9	174.0	9.3	6.93	7.7	341.64	10.9
Cropland	46.1	51.4	153.5	55.0	452.9	55.2	914.5	49.1	45.72	50.5	1612.62	51.3
Built-up	3.7	4.1	108.7	39.0	196.7	24.0	764.3	41.1	35.01	38.7	1108.35	35.3
**Total**	**89.6**	**100**	**278.9**	**100**	**820.8**	**100**	**1860.7**	**100**	**90.5**	**100**	**3140.6**	**100**

**Table 5:** Rate of LULC change in hectare per year.

LULC classes	Year		
	1986–2000	2000–2016	1986–2016
Forestland	−2.26	−0.66	−1.4
Plantation	28.41	−40.39	−8.28
Grassland	−0.57	−29.45	−15.97
Cropland	−29.27	10.11	−8.27
Built-up area	3.69	60.39	33.93
