# Supplementary material for: Data on spatiotemporal land use land cover changes in peri-urban Addis Ababa, Ethiopia: Empirical evidences from Koye-Feche and Qilinto peri-urban areas
Source: Data Brief. 2017 Apr 20;12:380–5. doi: 10.1016/j.dib.2017.04.018 (PMC5413857; doi:10.1016/j.dib.2017.04.018)
Supplement: Supplementary file 1 — Supplementary material [file mmc1.doc]

**Conflict of interest form**

There is no any conflict of interest in this data article.
